# Older Adults’ Experience of Meaning at the End of Life in Two Danish Hospices: A Qualitative Interview Study

**DOI:** 10.3389/fpsyg.2021.700285

**Published:** 2021-09-17

**Authors:** Dorte Toudal Viftrup, Christina Prinds, Ricko Damberg Nissen, Vibeke Østergaard Steenfeldt, Jens Søndergaard, Niels Christian Hvidt

**Affiliations:** ^1^Research Unit of General Practice, Department of Public Health, University of Southern Denmark, Odense, Denmark; ^2^Research Unit of Gynecology and Obstetrics, Department of Clinical Research, University of Southern Denmark, Odense, Denmark; ^3^Department for Research and Development, University College South Denmark, Haderslev, Denmark; ^4^Centre for Nursing, University College Absalon, Roskilde, Denmark; ^5^Academy of Geriatric Cancer Research (AgeCare), Odense University Hospital, Odense, Denmark

**Keywords:** meaning, hospice, end of life, concrete hope, absolute hope, interpretative phenomenological analysis, qualitative method

## Abstract

The aim of this study was to explore how older adults (aged > 65) confronted with imminent death express their thoughts and feelings about death and dying and verbalize meaning. Furthermore, the aim was to investigate how health professionals could better address the needs of this patient group to experience meaning at the end of life. The study applied a qualitative method, involving semi-structured interviews with 10 participants at two hospices. The method of analysis was interpretative phenomenological analysis. We found three chronological time-based themes: (1) Approaching Death, (2) The time before dying, and (3) The afterlife. The participants displayed scarce existential vernacular for pursuing meaning with approaching death. They primarily applied understanding and vocabulary from a medical paradigm. The participants’ descriptions of how they experienced and pursued meaning in the time before dying were also predominantly characterized by medical vernacular, but these descriptions did include a few existential words and understandings. When expressing thoughts and meaning about the afterlife, participants initiated a two-way dialogue with the interviewer and primarily used existential vernacular. This indicates that the participants’ scarce existential vernacular to talk about meaning might be because people are not used to talking with healthcare professionals about meaning or their thoughts and feelings about death. They are mostly “trained” in medical vernacular. We found that participants’ use of, respectively, medical or existential vernacular affected how they experienced meaning and hope at the end of life. We encourage healthcare professionals to enter into existential dialogues with people to support and strengthen their experiences of meaning and hope at the end of life.

## Introduction

This study focuses on how older people facing death at two Danish hospices experience meaning in the prospect of death. Denmark is one of the most secularized countries in the world – among researchers it is described as “the world’s least religious society” (Zuckerman, 2008). Studies also indicate that devout Christians in Denmark exhibit high degree of private and individualized religious faith (Viftrup et al., 2016, 2017; Nissen et al., 2019). Furthermore, studies have found that Danes generally have fewer spiritual or religious resources compared to less secularized societies. Therefore, spiritual and religious ways to experience meaning in life are less available (Ausker et al., 2008; la Cour, 2008; Pedersen et al., 2012). Despite this, studies have found that Danes increase their need for spiritual and religious meaning when facing severe illness or being confronted with death and dying (la Cour, 2005; Ausker et al., 2008). One study has also demonstrated a correlation between the severity of illness and the tendency to spiritual and religious thoughts and practices among Danes (la Cour, 2008). In recent years, the Danish State Church has set up the greatest number of support groups in the country related to grief and loss. This also indicates the relevance of religious and spiritual meaning among Danes. The State Church groups focus more on spiritual and religious aspects of meaning than do secular grief groups (Thomsen et al., 2014; Larsen, 2019). The preference among the general Danish population for Church-run groups indicates an increasing need in the population to find and express meaning in illness, loss, death, and crisis, through the use of spiritual and religious understandings and existential vernacular. However, due to the increased secularization and individualization of the Danish society, there is a paucity of knowledge on religious and spiritual meaning among Danes (Zuckerman, 2008; Stimpel and Raakjær, 2017). Pedersen et al. (2018) found religious characteristics to be more strongly associated with meaningfulness than socio-demographic variables in a study of Danish people’s personal meaning in life. Generativity was most strongly associated with meaningfulness, followed by spirituality, attentiveness, and explicit religiosity (Pedersen et al., 2018). The latter study was based on the Danish version of the Sources of Meaning Scale (SoMe-DA) in association with socio-demographic and religious characteristics. These studies indicate the relevance of religious and spiritual meaning among Danes and in other highly secularized countries, especially when facing severe illness and the prospect of death.

International studies also point to an increase in need for spiritual and existential meaning among older people, for whom death naturally becomes more present (Fleischer and Jessen, 2008). Furthermore, older people more often experience “existential loneliness” and “invisibility.” They have very few people to talk with about their thoughts on death, dying, and other existential dilemmas, and this increases their risk of isolation and depression (Heap, 2001). Several studies from different Europeans countries points out how older people don’t get sufficient help with their spiritual and existential concerns, care organizations had few active policies on dealing with older people’s existential questions, and professionals need training and appropriate qualifications to address older people’s existential loneliness related to existential aspects of aging and care (Harder and Friis, 2010; Erichsen and Büssing, 2013; Evans et al., 2014; van der Vaart and van Oudenaarden, 2018; Sundström et al., 2019). Both Danish and international studies suggest that terminally ill people can improve their quality of life by talking about their feelings and thoughts concerning death and the afterlife with healthcare staff (Strang et al., 2001; McClain-Jacobson et al., 2004; Murray et al., 2004; la Cour, 2008; Balboni et al., 2010). The European Association for Palliative Care (EAPC), in collaboration with the World Health Organization (WHO) European Office have developed a comprehensive list of learning goals that are essential to multidisciplinary postgraduate palliative care education within Europe (Paal et al., 2019). The EAPC points out the importance of spiritual care as an integral part of palliative care and, accordingly, suggests incorporating it into educational activities and training models in palliative care (Best et al., 2020). To improve palliative care for older people, for whom death and dying are naturally more present, knowledge of their experiences of meaning at the end of life seems highly relevant. One significant way, people experience spiritual and existential meaning is through feelings and experiences of hope (Graven and Olsen, 2018).

*Hope* is of particular importance for terminally ill people in the experience of meaning in dying (Owen, 1989; Nierop-van Baalen et al., 2016; Baczewska et al., 2019). A study showed how hope changes with disease progression. Initially, people hope for miraculous healing, but when treatment is no longer effective in curing, their hope changes and they start to prepare for the end of life (Daneault et al., 2016). A study on the meaning of hope among people with cancer in the palliative phase points to people using different strategies to increase their hope. This is described as the “the work of hope,” and increasing health professionals’ understanding of people’s “work of hope” may lead to better care and support (Nierop-van Baalen et al., 2016). A recent study on hope among terminal cancer patients found their highest levels of hope were in the spiritual-religious areas (e.g., hope in a deeper meaning), while the lowest levels of hope were related to finding a cure for the disease (Baczewska et al., 2019). Danish studies with terminally ill people indicate that religious and spiritual meaning is central to their well-being (Graven, 2015; Boelsbjerg, 2017, 2018). Several qualitative studies reveal that terminally ill people articulate spiritual notions and understanding about death and the afterlife. However, they rarely discuss these thoughts with the healthcare professionals (Moestrup, 2016; Moestrup and Hvidt, 2016; Boelsbjerg, 2018). Several studies have found that physicians and health professionals find it difficult to talk with patients about death, even when patients would like to (Singer et al., 1999; Wenrich et al., 2001; Wright et al., 2008; Assing Hvidt et al., 2016). A study suggests such hesitation could be because physicians are deeply rooted in a dominant medical paradigm characterized by a solution-focused medical vernacular, and because they are afraid of religious faith. This was their primary barrier to entering into spiritual and existential dialogs with people (Assing Hvidt et al., 2016). Furthermore, conceptualizations and vocabulary belonging to a medical paradigm are increasingly being implemented into the general language in secularized societies. This also seem to limit other ways for people to express meaning about what it means to fall sick, face a crisis, get older, or die (Illich, 1975; Bury, 2009; Hvas et al., 2011; Brinkmann and Petersen, 2015).

The present study qualitatively explores how older people confronted with imminent death express their thoughts and feelings about death and dying and how they verbalize meaning. This knowledge is highly relevant to improving healthcare and enhancing experiences of not only meaning and hope, but also quality of life at the end of life for older people. The aim of the study is to provide knowledge that may give health professionals better insight into older people’s need for meaning at the end of life, and how to address this need better.

## Materials and Methods

To study older people’s subjective thoughts and verbally articulated descriptions of their experiences of meaning while at a hospice at the end of life, a qualitative study was conducted. It included semi-structured interviews with older people at two hospices in Denmark. The research method and design applied were interpretative phenomenological analysis (IPA). IPA is a qualitative research method that uses a hermeneutic-phenomenological approach that focuses on how people perceive and make meaning of their life experiences or of the particular situations they face (Smith and Osborn, 2003, 2008; Assing Hvidt, 2018). IPA was therefore considered an appropriate method to study older people’s descriptions of their thoughts and experiences about meaning when facing death and dying.

### Setting and Participants

A convenience sampling strategy was applied (Farrokhi and Mahmoudi-Hamidabad, 2012; Palinkas et al., 2015). Interview participants were selected in close consultation with the hospices′ staff, based on considerations of the participants′ physical and mental resources to engage in interviews, and their ability to verbalize their thoughts and feelings. The vast majority of patients at the two hospices are older (aged > 65), ethnic Danes and their primary diagnosis is cancer (Sundhedsstyrelsen, 2011). However, variation in gender (six women and four men) was sought. The participants had various terminal cancer diagnoses and the mean age was 71. Based on ethical considerations, participants should meet the inclusion and exclusion criteria. The inclusion criteria were: older people (aged > 65) staying at one of the two hospices. They should voluntarily participate in the interviews, after receiving information about the study. The exclusion criteria were: Individuals suffering from a distorted perception of reality, severe cognitive or memory problems, or individuals who for other reasons were considered by the healthcare professionals to be physically or mentally too weak to participate in an interview. Interviews with the 10 participants took place in the participants’ own rooms and lasted between 19 and 56 min (average 29 min). All interviews were recorded and transcribed in an anonymized manner, showing only age, gender, marital status, cancer type, and time since terminal diagnosis. Audio files were thereupon deleted (Kvale and Brinkmann, 2009).

### Data Collection

Data collection took place from December 2017 to November 2018. The qualitative approach with 10 semi-structured interviews provided a good basis for insights and understanding into the subject from an IPA perspective. IPA has an idiographic focus and semi-structured interviews with deep and detailed first-person perspectives of the phenomenon are therefore a highly recommended data collection method. The interview guide centered on open-ended questions concerning the participants’ thoughts and feelings about death, dying, and meaning. The first and last authors were involved in establishing relationships with the hospices and designing the study. The interview guide was developed in collaboration with two groups of staff from each hospice and the first and last authors. Thereby, the interview guide was informed by the practical knowledge of hospice practice and the studies presented in the introduction. We were also interested in the participants’ verbalization of meaning, and therefore the interviewer would prompt the participants to elaborate on their thoughts. IPA requires an interviewer to have an empathetic attitude and the ability to facilitate a safe environment in which participants share their thoughts and feelings frankly and honestly. The interviews were conducted by the first author. She is a clinical psychologist with professional experience of loss and grief processes, and she received psychological supervision during the time of the interviews. Table 1 presents examples from the interview guide.

**TABLE 1:** Examples from the interview guide.

Open Ended Questions	Prompting Questions
As a patient, what do you often think about here at the hospice?	*Will you explain to me what concerns you the most these days?*
	*What does it feel like, for you, to be in your situation (here at the end of life)?*
Can you tell me what you think about death?	*Will you tell me more about how you feel when thinking about death (dying)?*
	*What do you think happens when you die/someone dies?*

### Data Analysis

In line with IPA’s idiographic focus, themes were developed inductively. However, IPA aims to account for both the important generic themes in an analysis as well as the individual experiences of life of the particular participant who has told their story. It operates at a level that is solidly grounded in the text and also moves beyond the text to a more interpretative and psychological level (Smith, 2004). The themes of this study are therefore further discussed and interpreted in relation to a broader theoretical perspective on *hope*. This theoretical framework will be presented further down in the text. The 10 interviews were thematically analyzed with a focus on participants’ descriptions of their thoughts about meaning at the end of their lives. Interviews were transcribed by the first author, and the meaning content was accentuated in the transcripts; speech sounds and repetitions were omitted, while pauses for thought and expressions of emotion were included, so that the transcripts emphasized the understanding of the participants’ statements and meanings.

The analysis followed the four steps recommended for an IPA analysis (Smith et al., 2009): The *first* step consisted of close reading of the 10 interviews; each interview was read several times and the authors wrote comments in the text where each participant had expressed particular understandings and thoughts about their experiences of meaning at the end of life. In the *second* step, we returned and reread each interview again, and attempts were made to rewrite the comments from the first step into more precise phrases about participants’ verbally expressed meanings about death and the afterlife. The comments from each interview were reformulated into incipient themes across all 10 interviews. In the *third* step, incipient themes were compared, and connections between them were explored to allow for a more analytical understanding of the themes. In the *fourth* step, we assigned the groupings of themes general names, and a structure emerged about participants’ experiences of meaning about death and the afterlife. Themes that were not firmly rooted across the 10 interviews, together with themes that were not considered relevant to the dominant structure, were deleted. It was the authors who assessed which themes should be eliminated and which should be focused on. Three overarching chronological time-based themes were generated: (1) Approaching Death, (2) The time before dying, and (3) The afterlife. In the fourth step, we also identified two ways through which the participants verbally expressed their experiences of meaning, which ran across the three main themes. These concerned how the participants talked of meaning about death with either medical or existential expressions and concepts.

### Ethical Considerations

Ethical principles for oral and written informed consent, voluntariness, anonymity, and confidentiality were followed, so that all participation took place in accordance with the ethical requirements of the Declaration of Helsinki (, 2013). The project was registered at SDU Research & Innovation Organisation (RIO) (notification number 10.467) and carried out in accordance with RIO’s guidelines and Danish legislation on managing personal data. Ethical implications of the project’s research activities and the special ethical implications in relation to patients in hospices (e.g., the participants would be dead by the time the study was completed) were continuously discussed among the authors.

### Rigor and Trustworthiness

A qualitative methodology was applied, as this seeks to increase depth and understanding of the subject under investigation (Kvale and Brinkmann, 2009). The goal of qualitative research is to produce a credible analysis and generate hypotheses, rather than provide a definite analysis (Bryman, 2001). The four qualitative criteria for validity, as presented by Yardley (2000), are widely used in and recommended for IPA studies, and these were addressed throughout the study: (1) *Sensitivity to context* was maintained by immersive and disciplined attention to the accounts of the participants; (2) *commitment and rigor* were sought through involvement and collaboration between authors, participants, and staff at the hospices at all points during the study; (3) *transparency and coherence* were upheld by describing the different stages of the research process; and (4) *impact and importance* were sought by emphasizing how the study should tell the reader something interesting, important, or useful.

The first author conducted the interviews while being supervised on personal feelings, interview approach, and data saturation by both another psychologist and the second and last authors. The analysis was performed by the first, second, and last authors. Furthermore, to ensure reliability in IPA studies, participants are often involved in validating the researchers interpretations. The staff at the hospices participated in validating the themes on behalf of the 10 participants, who had all died by the time the analysis was conducted. This validation process led to discussions on *hope* in relation to meaning at the end of life, which inspired the theoretical discussion of this manuscript. All authors were involved in discussing the data, analysis, and writing of the manuscript.

In order to keep awareness of the various motivations, interests, goals, personal beliefs, values, and preconceptions of researchers as well as the hospices, the first author wrote notes at all stages of the research process and discussed these with the other authors. Authors were aware of being as transparent as possible about their own preconceptions concerning death, dying, religiosity, spirituality, and meaning and about being embedded in a specific caring paradigm. The authors represented six different fields within healthcare: Psychology, midwifery, psychiatry, nursing, general practice, and theology.

The software program NVivo version 12 was used to manage and structure the data material.

### Theoretical Framework

For this study, we use the definition of meaning in life as presented by Tatiana Schnell (Schnell, 2009, 2021): *Meaning in life can be determined as the direction – or purpose – that someone pursues, and the ensuing subjective and dynamic evaluation of their life as more or less meaningful”* (p. 6). Furthermore, we differentiate between whether the interviewed participants expressed their pursuit of meaning in a medical or existential vernacular. We understand the notion of medical vernacular as the verbalization of human challenges and life circumstances primarily as medical conditions that must be understood, examined, prevented, or treated medically and physically (Illich, 1975). We define existential vernacular as the use of verbalization, concepts, and understandings within three existential domains of meaning in a framework of existential meaning-making. *The secular domain* encompasses relationships with aspects of life that are meaningful to the individual but are not religious, e.g., values or meaning of family or work. *The spiritual domain* represents the transcendent, inner spiritual life experienced by the individual. *The religious domain* covers beliefs and understandings that are shared and practised with others, e.g., bible reading or church attendance (la Cour and Hvidt, 2010).

To discuss and interpret how our older participants experienced and verbalized meaning of death and dying, the notion of hope was included. This theoretical framework was not included in the interview guide, as the notion of hope first came to attention during the validation process with the staff from the two hospices. Another Danish hospice study, by Vibeke Graven (2015), addressed hope as a concept for understanding how terminally ill people experience meaning at the end of life. It became clear that existential and medical vernacular, respectively, affected people’s experiences of hope as a source of meaning (Graven, 2015). Graven’s study presents the analysis of hope by French existentialist philosopher and theologian Gabriel Marcel (Marcel, 1951). Marcel distinguished between desire and hope. *Desire* is when one wants or seeks something in particular. Desire is the concrete hope that looks to the near future. *Hope*, however, is an open-ended expectation in which one anticipates without knowing exactly what it is one is waiting or hoping for. Hope is not something one can dictate or create by oneself alone. Rather, it is a grace which one receives. Marcel explains: “The only genuine hope is hope in what does not depend on ourselves, hope springing from humility and not from pride” (p. 32).

In this understanding, the medical vernacular is based on *desire*, e.g., hoping to get well, to have a good day in spite of pain, or to be able to die in one’s own home. *Desire* represents people’s concrete hopes for the near future, and it can bring stability, joy, or meaning to life. However, desire is also conditioned by external circumstances and can therefore easily be disappointed (Graven, 2015; Graven and Olsen, 2018). Conversely, *hope* is of a transcendent and existential nature that embraces man’s whole existence and being, and therefore it is called “the absolute hope” (Marcel, 1951; Knox, 2003). *Hope* has a metaphysical, relational, and existential significance that extends beyond death and external circumstances. Marcel describes this as “the mystery of hope” and explains that the absolute hope comes to humans when opening oneself to being (Marcel, 1951; Knox, 2003). In her study at hospices, Graven describes Marcel’s concept of absolute hope as the “hope of being” (Graven, 2015; Graven and Olsen, 2018). She presents three forms of hope of being: (1) The hope of love, (2) The hope of universal emotions, and 3. The hope of an afterlife. *The hope of love* is of an ethical, relational nature and is expressed through acts of love and charity in relationships between people. These actions become hopeful because they express a love that cannot die but, rather, transcends earthly life. *The hope of universal emotions* derives power from aesthetic expressions, such as nature, art, music, and traditions and it can open up to universal and shared emotions. *The hope of an afterlife* can be a religious hope of an afterlife, which involves beliefs in a personal God. It can also entail more individualized notions of an afterlife, where the individual finds hope in an eternity dimension that transcends earthly life (Graven, 2015).

Marcel’s distinction between *concrete hope (or desire)* and *absolute hope*, and Graven’s elaboration on the hope of being, seem particularly relevant when exploring how older people experience meaning at the end of life.

## Results

Table 2 presents summarized information about the participants This information has been obtained during the interviews with the participants.

**TABLE 2:** Participants information.

	Age	Gender	Dominating Cancer Type	Marital Status	Time Since Terminal Diagnosis
1.	65	Female	Kidney and liver cancer	Single	3–4 months ago
2.	67	Male	Leukemia	Married	6–8 weeks ago
3.	85	Female	Stomach	Widowed	3 months ago
4.	72	Female	Lung/breast	Widowed	2.5 months ago
5.	65	Male	Leukemia	Single	3 weeks ago
6.	69	Male	Lung cancer	Married	8–9 weeks ago
7.	72	Male	Lung cancer	Widowed	6–8 months ago
8.	69	Female	Breast cancer	Cohabiting	Approx. 2 years ago
9.	66	Female	Stomach cancer	Married	6 weeks ago
10.	71	Female	Leukemia	Divorced	2–3 months ago

### Approaching Death

The first theme, *Approaching death*, is characterized by participants trying to comprehend that they are dying from their disease. In the interviews, the participants explained how they received information at the hospital that curative treatment was no longer possible. For most of the participants, this message was given after a long treatment trajectory; some had been in and out of hospitals and received a range of treatments for years. Statements such as: *“It was the doctor who told me that there was nothing more that could be done for me*…” *(No. 4)* are found repeatedly in the data material. It was during these situations the participants became aware that their death was imminent. Their verbalization and descriptions in relation to pursuing meaning in those situations were characterized by an extensive medical vernacular:

*“They gave me seven treatments to beat it, but it turned out that it could not cure it. On the contrary, the metastasis had instead grown quite a lot. Then they stopped that treatment, and there was only one more bullet left in the gun, and that was chemo. I was given that until recently. I was at a control scan last week and then I got the answer on Monday*… *the chemo had not changed anything, on the contrary the cancer had spread a lot more. And yes, then they gave up the treatment. There is nothing more to do. It was of course a hard message to receive, and yes, I would have liked to have had a few more years*…*” (No. 5).*

This quote illustrates how the participants needed to feel that their life and death had some meaning when approaching death. They described the treatment trajectory and gave medical details about no longer being able to receive curative treatment. It was recurrent across interviews that participants began by describing the overall course of the disease in medical terms when they were asked about their thoughts on death. Their descriptions of their disease trajectory were detailed and exhaustive with comprehensive use of several medical words and understandings. However, in these descriptions about their meaning about death, participants would also include a short comment of a more existential nature, most often as a concluding comment, like the one in the quote above: “*It was of course a hard message to receive, and yes, I would have liked to have had a few more years*…*”(No. 5).* Although they were prompted to elaborate on these comments of more existential nature during the interviews, it was characteristic that these elaborations remained quite brief and concise, such as: *“Yes, you always want more years, right*…*” (No. 8)* or *“No, it was not what I was hoping to hear*…*”(No. 5)* or *“It was difficult to hear*…*”(No. 2).*

### The Time Before Dying

The second theme, *The time before dying*, concerns how participants talked about their thoughts about the final time, up until death occurs, and how they verbally express meaning at the end of life. The theme involves both the participants’ thoughts on the very last minutes or hours before death occurs as well as the present time, their thoughts on being in the hospice right now and up until they would die. In their descriptions we again found a predominant use of medical vernacular to describe the existential experiences of dying:

*“When dying, I am afraid that I would just lie there in bed with huge pain*… *or get suffocated*… *because I now have cancer in my lungs, and that feeling of suffocation*… *If that’s how you feel when*… *the idea of that makes me feel anxious, but if it’s just that like you feel more and more sleepy, and then in the end you do not open your eyes again. Then I think it’s ok*…*”(No. 4).*

This participant talked about her thoughts on the last minutes or hours before death. Verbally, she mainly expressed concerns related to the physical aspects of dying. However, the participant primarily applied medical vernacular to express her thoughts. It was recurrent across the interviews with the 10 participants, that when describing these kinds of thoughts, they talked extensively about the medical/physical aspects of dying. Despite this, however, it was found that they mixed existential words and understandings into the dominating medical vernacular. They did this to a greater extent than when expressing how they approach. This is for example seen in the above quote, where, in between the predominantly medical vernacular the participant talks about “feeling anxious,” “not opening your eyes again,” and “thinking it is ok.” This may be because the participants are talking about the time before dying in the present time, whereas approaching death was expressed in the past tense.

There was one interview that differed from the others. This participant used a predominantly existential vernacular when describing her thoughts on the time leading up to death.

*“Of course, I would rather live but that’s not how God intended it. So now I’m here, and I can feel it in my body day by day which way it goes now*… *So, I spend the days here (at the hospice) turning away from this world and bidding this world goodbye and turning to God’s eternity. It’s a bit of a process*…*”(No. 1).*

This participant used existential vernacular to describe her religious meaning and idea of what dying meant to her. None of the other nine participants used existential vernacular with religious understandings to the same extent, despite some of them also displaying other forms of religious beliefs or spirituality. The participant further elaborated on her faith being helpful in pursuing meaning during the time leading up to dying:

*“Of course, I am sad sometimes [*…*] but this is what has become my lot. Yes, then I place it in God’s hands and say: Only in the hope in God, my soul is quiet. And the soul has really become quiet, and the hope has grown big, but that does not mean that there are no dark moments*… *There certainly are. But God gives you power in the midst of it all to bear it, and I do not know how I could handle this if I did not have an eternal God with eternal arms reaching out to me. And he really does that quite literally.*” (*No. 1).*

This participant had an extensive and detailed existential vernacular for religious meaning. This may be explained by her having practised the Christian faith most of her life and thereby learning “to talk religiously” in interaction with other Christians and in church. However, several of the other participants also displayed religious and/or spiritual understandings in their pursuit of meaning. These were characterized by being expressed forms of spiritual/religious practices, as this quote shows:

*“*… *This (shows a stone with a spiritual text printed on it), I hold it and hug it every morning when I wake up*… *so I do believe in something*… *I have never been one who has been much of a church goer, but I have always believed in something anyway*… *I also had a visit from our own chaplain at home before I came down here (to the hospice).” (No. 9).*

This participant used existential vernacular to describe a meaningful spiritual practice in which she handled her thoughts and concerns about dying. However, just prior to this quote, the participant had described in great detail with medical vernacular how she was ensured medical pain relief up until the time of death. She had thoroughly explained and showed the interviewer how the morphine pump worked.

### The Afterlife

The third theme: *The afterlife* is characterized by the participants’ describing their hopes about and beliefs in what happens after death has occurred. The theme primarily derived from the participants’ answers to the question: “What do you think happens when you die/someone dies?” It was apparent across the interviews that participants answered this question and talked about this topic with a predominantly existential and far less medical vernacular.

An obvious explanation for the dominant existential vernacular among the participants when talking about the afterlife may be found in the fact that medical science has very little to say about that. Therefore, the medical vernacular is limited or not relevant when discussing perceptions or ideas about what may happen after death. However, the question could also be answered in the medical vernacular related to the dying body, such as: The heart stops beating, the organs set out, the cancer won, it becomes hard to breathe, or similar examples of medical concepts of what happens when you die. However, none of the participants responded with any notions of medical vernacular; instead, their answers reflected existential thoughts and understandings about the afterlife, regardless of whether they affirmed or denied any belief in an afterlife. Furthermore, a change in the form of communication for the interview also became apparent. When answering the question about what might happen when death occurs, the participants would initiate more dialog with the interviewer, instead of just being the one answering questions. For example, one participant said:

*“I believe in God, but it’s not that I think that you resurrect in a new way. I think when I die, I just sleep*… *I just stay asleep next to [name of the late spouse] and then we continue to sleep together. I do not believe anything else but that [.] we did, and I still do, when I fall asleep every night, I always say; thank you for this day I’ve had. But it’s not like I’m religious and go to church and*… *I don’t, but I still believe that there is something spiritual, but you cannot know what*…*What do you think happens?”(No. 3).*

The quote is an example of how the participants would also begin asking the interviewer about her thoughts concerning the afterlife. When asking them about their thoughts on what happens when death occurs, the participants would initiate a change from mainly a one-way interview to a dialog between participant and interviewer. This was recurrent across the interviews. The interviewer (first author) felt it essential to respond to this initiative by entering into the dialog with her own thoughts, beliefs, and doubts about the afterlife. Although she was still focused on exploring the participants’ perspectives in the dialog, she experienced that the participant incited sincerity in her response as well as a genuine dialog. For example, she responded to the above question with: *“I also think there is something*… *I also really hope so”* and the participant interrupted her: *“Oh, you are still young [*…*] but perhaps we’ll meet again*… *(both laughing)” (No. 3)*. They continued talking about their thoughts, hopes, beliefs, doubts, and concrete notions of what actually might happen when they (both participant and interviewer) would die; it became a dialog where they shared their mutual thoughts, concerns, and notions about the afterlife. The interviewer entered into a pursuit of meaning about the afterlife together with the participant, where they together used existential vernacular.

The participants expressed varied thoughts and meanings about the afterlife, ranging from religious understandings to individual spiritual conceptions, to atheistic notions that, however, were more reminiscent of agnosticism. None of the 10 participants actually expressed a form of atheism, in which they fully wrote off notions of God or a transcendent understanding of an afterlife.

Furthermore, it was characteristic of all of them that their descriptions and meanings about the afterlife were rooted in their individual and specific life circumstances, as the example above also shows. This participant’s (no. 3) thoughts about the afterlife were rooted in her life circumstances with a deceased spouse, and having lived without any particular concern with religion and church attendance. She primarily pursued meaning, applied existential vernacular, and related to the unknown of death and dying through the life circumstances already at hand. For this participant (no. 3), it is important to note that her use of existential vernacular seemed to bring her comfort in terms of experiencing meaning about the afterlife. However, not all participants’ thoughts about the afterlife were comforting to them. For example, another participant explained:

*“I do not know what I believe, but I believe in something*… *I have always said that there is something*… *there is something after death*… *my youngest son he says: “Non-sense”*… *and then I say: “But that’s my faith, I’m allowed to have it”*… *and yet I also say to myself that I also know very well*… *because I have seen*… *that we have had many in the family who have died from cancer*… *well, I know that when they are gone, then they are no longer here*… *but therefore I still cannot really understand it, and then I still think that there must be*… *there must be something*… *so I have a faith*…*”(no. 9).*

During the interview, this statement was followed up by a question about whether she had some more specific thoughts about this “something,” and she responded: *“No, I just do not think that*… *no, I just do not believe*… *it cannot be that there is nothing and that you just*… *(cries)*…*” (No. 9)*

This participant also asked the interviewer about her thoughts and beliefs on what happens when you die and initiated a dialog about the topic. She asked directly: *“Do you think that there is something*… *when I die?”* and the interviewer (first author) replied: *“Yes, I think there is something*…*”* and then they had an emotional and genuine dialog about the possibility of an eternity and their feelings of both hopes and doubts.

It became clear that this participant also expressed thoughts and meanings about the afterlife and related to the unknown of death through her life circumstances already at hand. However, her life circumstances involved a son, who apparently did not believe in an afterlife, as well as circumstances with other family members who had died from cancer. The existential vernacular, she applied in pursuing meaning about the afterlife, stemmed from these life circumstances. However, contrary to the other participant (No. 3) whose quotation was presented above, this participant’s (No. 9) existential vernacular and experiences of meaning about the afterlife did not seem to be of much comfort to her.

### Theoretical Interpretation: Concrete Hope, Absolute Hope, and Meaning

Studies suggest that participants’ perception of hope changes with disease progression (Daneault et al., 2016). In this study, we saw a change in participants’ use of, respectively, medical and existential vernacular that follows their temporal realization of approaching death (theme 1), the time before dying (theme 2), and the afterlife (theme 3). The participants’ predominant use of medical vernacular to describe their experiences and meaning of death becoming imminent (theme 1) may be due to the fact that, in connection with the course of their illness, they have primarily *desired* to recover from the illness. The *concrete hope* seems to relate well with the medical paradigm and vernacular, where their challenges are seen as a physical condition that can be “solved” (Illich, 1975). The *concrete hope* is related to desiring that the medical treatment have a positive and curative effect. In contrast, the *absolute hope* is characterized by not being solution-focused; instead it implies aspects of faith and trust that extend beyond death and external circumstances (Marcel, 1951). The *absolute hope* contrasts with the medical paradigm and vernacular. Healthcare professionals embedded within a medical paradigm might primarily tend to communicate the *concrete hope* in their dialog with people, instead of using existential vernacular that could facilitate the *absolute hope* for people.

Despite the fact that hospice residents usually no longer hope for survival from their illness, the participants’ descriptions of meaning in relation to the time before dying (theme 2) were still characterized by *concrete hope*. For example, we found that they expressed *concrete hope* of a painless death and to just sleep into dying. When the participants predominantly used medical vernacular to talk about the time before death occurs, this indicates that they were primarily concerned about the medical and physical aspects of dying. In that light, it becomes essential for healthcare professionals to talk with dying people about medical and physical aspects of dying. Studies found that, even in conversations with hospital chaplains, much of the conversation concerned what you eventually die from and how death comes about in practical terms (Strang and Strang, 2002). However, it may also be, as previously explained, that participants are more “trained” in medical rather than existential vernacular.

Graven (2015) found in her study that dying people exhibit both *concrete hope* and *absolute hope*. People benefit from speaking with healthcare professionals with a mixture of the two forms of hope and using both medical and existential vernacular (Graven, 2015). In the present study, we did not find that the dying older people mixed the two forms of hope to a significant extent. Instead, we found that their vernacular changed as time went on, where they moved from primarily medical vernacular and *concrete hope* to existential vernacular and *absolute hope*. The *hope of the afterlife* became particularly present and relevant for the participants’ thoughts and meaning about afterlife (theme 3).

Whether the participants’ experience of meaning and hope were comforting to them, depended on their life circumstances; for example, a deceased spouse, a son who did not believe in the afterlife, or healthcare professionals who may or may not enter into an existential dialog about the afterlife.

All the three types of absolute hope, the *hope of love*, the *hope of universal emotions*, and the *hope of an afterlife* (Graven, 2015; Graven and Olsen, 2018) are at stake when people experience meaning, by talking about eternal rest with a beloved spouse, focusing on God’s eternity, hugging a stone that has a spiritual text written on it, or initiating an existential dialog about the afterlife. We found that, when the participants’ life circumstances did not support meaning and hope, they may end up not experiencing hope in the face of the unknown of death. For example, the son who thinks that meaning about the afterlife is inconsequential may prevent his mother from the *absolute hope*, or a healthcare professional who only offers *concrete hope* in relation to a solution-focused medical paradigm may also hinder people’ experience of meaning and hope at the end of life.

## Discussion

Overall, our study supports such an assertion that being faced with death can lead to an intensification of spiritual, and/or religious considerations (Thune-Boyle et al., 2006; Jones et al., 2010), need for meaning (Fleischer and Jessen, 2008), and that spiritual and religious thoughts and practices may become more prominent (la Cour, 2008). Based on the present study, it is difficult to assess whether it also increased participants’ quality of life to talk about their thoughts and meaning about death and the afterlife with health professionals (Strang et al., 2001; McClain-Jacobson et al., 2004; Murray et al., 2004; la Cour, 2008; Balboni et al., 2010). However, our analysis indicates that it is important that participants’ life circumstances (including relatives and health professionals at hospice) support their existential vernacular and meaning. Furthermore, the participants in our present study used religious and/or spiritual elements in their existential vernacular, in particular when talking about their thoughts and meanings about the afterlife. This points to religious and spiritual concerns and feelings being central to the pursuit of meaning when faced with death and dying, even in a secular culture (Graven, 2015; Boelsbjerg, 2017; Boelsbjerg, 2018).

### Existential and Medical Vernacular

We found that our participants used scarce existential vernacular in the pursuit of meaning at the time when death was first acknowledged as imminent (theme 1) and they primarily applied understandings and vocabulary belonging to a medical paradigm (Illich, 1975; Bury, 2009; Hvas et al., 2011; Brinkmann and Petersen, 2015). The participants’ descriptions of how they experienced and pursued meaning in the time before dying (theme 2) were also predominantly characterized by the medical vernacular, but these descriptions did include a few existential words and understandings. However, there was no indication of this being caused by a high degree of privacy of thoughts and concerns about religious or spiritual issues as found in other studies (Andersen and Lüchau, 2011). All participants expressed themselves openly about their thoughts on death and dying. We did not find that the participants were reluctant to talk about their thoughts and meaning about death and dying, despite making little use of existential vernacular.

It seemed that participants in general applied their “most trained vernacular” to talk about death and meaning. They have learned to master this best after long courses of treatment and conversations with health professionals who primarily talk in the medical vernacular and are embedded within a solution-focused medical paradigm. This is also supported by the religious participant, who, unlike the other participants, used a predominantly existential vernacular, because she was highly trained in that. However, we found an increased use of existential vernacular and participants initiating two-ways dialog instead of continuing the one-way interviews when talking about the afterlife (theme 3). To discuss their thoughts and meaning concerning the afterlife, it seemed that the participants needed existential vernacular. However, because their existential vernacular was scarce, they also needed relationship and dialog to “train” the existential vernacular and to express meaning and hope that could not be conceived within medical vernacular.

The 10 participants expressed diverse meaning concerning the afterlife (theme 3). These were religious, individual, spiritual, as well as atheistic/agnostic notions and understandings. Their descriptions of their meaning about the afterlife and relating to the unknown of death were characterized by being relational and existential as well as rooted in their life circumstances. It was seen that the existential vernacular was comforting for some participants but not for others. This seemed to depend on whether the participant’s life circumstance supported his or her existential vernacular and experience of meaning about the afterlife.

## Implications

Health professionals may have an important role to play in strengthening people’s experience of meaning and hope when facing death (Graven and Olsen, 2018). An important aspect of caring for dying people may be to support and strengthen the person’s *absolute hope* in the face of the unknown of death, especially by entering into an existential dialog with people, and by being aware of not primarily using the medical vernacular when taking with terminally ill people.

Furthermore, findings indicate that participants’ existential vernacular had a much more relational and dialogical foundation. Relational and dialogical aspects of communication seem to be particularly relevant for people when expressing and pursuing meaning about death and the afterlife. However, this points to further difficulties for the healthcare professionals when engaging in existential dialog, as they tend to be deeply rooted in a solution-focused medical vernacular. To engage in a dialog about the afterlife, in the way that the interviewer did with the participants, might require a mutual sharing of thoughts, concerns, hopes, and doubts about the afterlife. Therefore, the study also points to a need for an increased focus on communication skills and critical reflection upon the dominant medical paradigm in current healthcare educational programs, as well as focus of further research on how specific communication affect patients’ well-being at the end of life. Further research in how dominating vernacular within healthcare is maintained and affects people, as well as research in the effect of dialogical communication are warranted. Hoping in some sort of a spiritual or religious realm was also found to be central to the participants’ experience of meaning (Baczewska et al., 2019). The existential vernacular seemed to support their religious and/or spiritual thoughts, beliefs, hopes, and meaning. It is found that quality of life increases when people are given better opportunities to express their thoughts, feelings, and meaning about the unknown of death (Balboni et al., 2010). This study suggests that healthcare professionals also should apply existential and not just medical vernacular when addressing terminally ill people, because this may strengthen their experience of meaning and hope in the face of death. Furthermore, this also emphasizes the importance of spiritual care as an integral part of palliative care and that it is important to incorporate it into educational activities and training models in palliative care, as recommended by the EAPC (Best et al., 2020).

## Limitations

In this study, we sought to examine older dying people’s subjective descriptions of their experiences of meaning in death and dying. Even though the length of the interviews considered the physical condition of the participants, we believe the qualitative approach with 10 semi-structured interviews provided a good basis for insights and understandings of a qualitative nature. The participants were selected by hospice staff, which may be a weakness of the study. To obtain a nuanced and complete data base, a strategic selection of participants could have been desirable (Patton, 1990). We have tried to ensure that the participants represented variation in gender and marital status, and as the participants of this study all are older (>65), ethnic Danes diagnosed with cancer, they are representative of people at Danish hospices (Sundhedsstyrelsen, 2011). Furthermore, IPA is explicit that homogenous samples work best in conjunction with its philosophical foundations and analytical processes (Smith et al., 2009). However, the socio-demographic characteristics of the sample reduce the transferability of the findings to other contexts, apart from Danish hospices. On the other hand, the findings related to how the participants’ use of, respectively, the medical or existential vernacular affected how they experienced meaning and hope at the end of life might be relevant for anyone faced with death and dying in secularized societies in which there is a highly dominant medical paradigm.

The research method of the study stems from IPA, which emphasizes description and interpretation of the data material. IPA moves from the participants’ subjective descriptions to a more theoretical level of interpretation, which depends on the authors’ interpretations and choice of theory. This means that the identified themes reflect the authors’ interpretations and that aspects of participants’ experiences on meaning may have been overlooked.

Many of the participants knew the interviewer as a psychologist. Being a clinical psychologist could be both an advantage and a disadvantage: An advantage, in the sense that her psychological knowledge and skill set could deepen her insights into and sensitivity for the life world of the participants; A disadvantage in the sense that a psychological interpretation of the subject matter could dominate and constitute bias and blind spots when analyzing the data. The other authors paid attention to and sought to avert such disadvantages and limitations.

However, our focus was the participants’ perspectives and descriptions of meaning about death and dying. Especially in relation to hope, we find that the results call for further research, which also involves an exploration at how healthcare professionals may sense and support different forms of hope among dying people.

For brevity, we have chosen to limit the theoretical framework of this study to the notion of hope as a specific approach to experiencing meaning, but it is obvious that concepts such as dignity or “the good death,” seen in similar studies, could also be included. In healthcare practice with dying people, these concepts can also be expected to be influenced by, respectively, existential and medical vernacular.

Further limitations could include the researcher’s approach to interviewing. She experienced it essential to respond to the participants’ initiative to enter into a dialog about the afterlife (theme 3). She entered the dialog with her own thoughts, beliefs, and doubts about death and dying. Thereby, she influenced the participants’ thoughts and expressions. On the one hand, her existential input in the dialog may have resulted in the increased existential vernacular of the third theme, which might not fully reflect the participants’ experiences of meaning. On the other hand, her relational sensitivity and response during the interview may also have contributed to new understanding about the relational foundation of existential vernacular, and the participants’ experiences of meaning about death may otherwise not have come to our knowledge.

## Conclusion

When studying how older people experience meaning about death and dying, we found three chronological time-based themes: (1) Approaching Death, (2) The time before dying, and (3) The afterlife. The participants expressed meaning variously through these three themes. They verbally expressed their experiences of meaning in two ways: respectively, in the medical and the existential vernacular. The participants would used great amounts of medical and low amounts of existential vernacular to express meaning about dying. However, when talking about the afterlife (theme 3), the participants used more existential notions and they also initiated a dialog with the interviewer.

Themes were discussed through an theoretical framework with the notion of hope. It seemed that participants’ use of vernacular affected how they experienced meaning and hope at the end of life. The *concrete hope* seems to be communicated through medical words and focus, whereas *absolute hope* was articulated through dialog that used existential vernacular. The study points to the importance of spiritual care (including strengthening communication skills and facility with existential vernacular) as an integral part of palliative care and current healthcare education.

## Data Availability Statement

The dataset, both the transcripts and the analysis based on the four steps recommended in IPA for this study can be made available on request to DV, dviftrup@health.sdu.dk.

## Ethics Statement

The studies involving human participants were reviewed and approved by RIO SDU (notification number 10.467). The patients/participants provided their written informed consent to participate in this study.

## Author Contributions

DV conducted the study and drafted the work. NH was involved in establishing relationships with the research field and designing the study. DV, CP, and NH coded and analyzed the data. All authors ensured reliability in the themes, revised and discussed the final manuscript, contributed to the article, and approved the submitted version.

## Conflict of Interest

The authors declare that the research was conducted in the absence of any commercial or financial relationships that could be construed as a potential conflict of interest.

## Publisher’s Note

All claims expressed in this article are solely those of the authors and do not necessarily represent those of their affiliated organizations, or those of the publisher, the editors and the reviewers. Any product that may be evaluated in this article, or claim that may be made by its manufacturer, is not guaranteed or endorsed by the publisher.
